# The effects of temperature and exercise training on swimming performance in juvenile qingbo (*Spinibarbus sinensis*)

**DOI:** 10.1007/s00360-012-0690-7

**Published:** 2012-08-18

**Authors:** Xu Pang, Xing-Zhong Yuan, Zhen-Dong Cao, Shi-Jian Fu

**Affiliations:** 1 College of Resources and Environmental Science, Key Laboratory of Southwest Resource Exploitation and Environmental Disaster Controlling Project of the Education Ministry, Chongqing University, Chongqing, 400030 China; 2 Laboratory of Evolutionary Physiology and Behaviour, Chongqing Key Laboratory of Animal Biology, Chongqing Normal University, Chongqing, 400047 China

**Keywords:** Exercise training, *Spinibarbus sinensis*, Swimming performance, Temperature

## Abstract

To investigate the effects of temperature and exercise training on swimming performance in juvenile qingbo (*Spinibarbus sinensis*), we measured the following: (1) the resting oxygen consumption rate $\left( {{\dot{\text{M}}\text{O}}_{{ 2 {\text{rest}}}} } \right)$, critical swimming speed (*U*_crit_) and active oxygen consumption rate $\left( {{\dot{\text{M}}\text{O}}_{{ 2 {\text{active}}}} } \right)$ of fish at acclimation temperatures of 10, 15, 20, 25 and 30 °C and (2) the $\dot{M}{\text{O}}_{{ 2 {\text{rest}}}}$, *U*_crit_ and $\dot{M}{\text{O}}_{{ 2 {\text{active}}}}$ of both exercise-trained (exhaustive chasing training for 14 days) and control fish at both low and high acclimation temperatures (15 and 25 °C). The relationship between *U*_crit_ and temperature (*T*) approximately followed a bell-shaped curve as temperature increased: *U*_crit_ = 8.21/{1 + [(*T* − 27.2)/17.0]^2^} (*R* ^2^ = 0.915, *P* < 0.001, *N* = 40). The optimal temperature for maximal *U*_crit_ (8.21 BL s^−1^) in juvenile qingbo was 27.2 °C. Both the $\dot{M}{\text{O}}_{{ 2 {\text{active}}}}$ and the metabolic scope (MS, $\dot{M}{\text{O}}_{{ 2 {\text{active}}}} - \dot{M}{\text{O}}_{{ 2 {\text{rest}}}}$) of qingbo increased with temperature from 10 to 25 °C (*P* < 0.05), but there were no significant differences between fish acclimated to 25 and 30 °C. The relationships between $\dot{M}{\text{O}}_{{ 2 {\text{active}}}}$ or MS and temperature were described as ${\dot{\text{M}}\text{O}}_{{ 2 {\text{active}}}} = 1,214.29/\left\{ {1 + \left[ {\left( {T - 28.8} \right)/10.6} \right]^{2} } \right\}\;\left( {R^{2} = 0.911,\;P < 0.001,\;N = 40} \right)$ and MS = 972.67/{1 + [(T − 28.0)/9.34]^2^} (*R* ^2^ = 0.878, *P* < 0.001, *N* = 40). The optimal temperatures for $\dot{M}{\text{O}}_{{ 2 {\text{active}}}}$ and MS in juvenile qingbo were 28.8 and 28.0 °C, respectively. Exercise training resulted in significant increases in both *U*_crit_ and $\dot{M}{\text{O}}_{{ 2 {\text{active}}}}$ at a low temperature (*P* < 0.05), but training exhibited no significant effect on either *U*_crit_ or $\dot{M}{\text{O}}_{{ 2 {\text{active}}}}$ at a high temperature. These results suggest that exercise training had different effects on swimming performance at different temperatures. These differences may be related to changes in aerobic metabolic capability, arterial oxygen delivery, available dissolved oxygen, imbalances in ion fluxes and stimuli to remodel tissues with changes in temperature.

## Introduction

Temperature is one of the most important abiotic factors in the habitats of ectothermic animals and has been called the ‘ecological master factor’ for animals (Brett 1971). As ectotherms in natural water bodies, fish are subjected to large diurnal and seasonal changes in temperature (Claireaux et al. 2006). Consequently, temperature has profound effects on certain important physiological functions and fitness-determining traits, such as growth, metabolism and swimming performance (Claireaux et al. 2006; Pang et al. 2011). Swimming is an important physiological activity and a survival-determining function for fish because it is closely related to food capture, predator avoidance and reproductive behaviour. Critical swimming speed (*U*_crit_, i.e. the water velocity at which a fish can no longer maintain its position in an incremental velocity test) is a widely used parameter for the evaluation of swimming performance in fishes (Reidy et al. 2002; Lee et al. 2003a; Farrell 2008). *U*_crit_ primarily reflects aerobic swimming performance; however, *U*_crit_ also includes anaerobic metabolism for some fish species (Lee et al. 2003a). Metabolic rate is a measure of the energy utilisation of an organism and is traditionally measured as the rate of oxygen consumption $\left( {{\dot{\text{M}}\text{O}}_{2} } \right)$. Previous research has indicated that the resting oxygen consumption rate $\left( {\dot{M}{\text{O}}_{{ 2 {\text{rest}}}} } \right)$ increases significantly with an increase in temperature (Clarke and Fraser 2004; Pang et al. 2010; Zeng et al. 2010). Studies of the relationship between temperature and swimming performance have found that plots of both *U*_crit_ and the active oxygen consumption rate ($\dot{M}{\text{O}}_{{ 2 {\text{active}}}}$, the maximum $\dot{M}{\text{O}}_{2}$ during the *U*_crit_ test) over a temperature range resemble bell-shaped curves for certain fishes (Hammer 1995; Lee et al. 2003b; Fangue et al. 2008; Zeng et al. 2009). However, other studies have identified positive correlations between temperature and both *U*_crit_ and $\dot{M}{\text{O}}_{{ 2 {\text{active}}}}$ (Schurmann and Steffensen 1997; Claireaux et al. 2006). In addition to $\dot{M}{\text{O}}_{{ 2 {\text{active}}}}$, the metabolic scope (MS, $\dot{M}{\text{O}}_{{ 2 {\text{active}}}} - \dot{M}{\text{O}}_{{ 2 {\text{rest}}}}$) is an important parameter for the evaluation of aerobic metabolic capacity (Claireaux et al. 2000; Lee et al. 2003b; Pang et al. 2011). In this study, we investigated the effects of a range of temperatures (10, 15, 20, 25 and 30 °C) on *U*_crit_, $\dot{M}{\text{O}}_{{ 2 {\text{active}}}}$, $\dot{M}{\text{O}}_{{ 2 {\text{active}}}}$ and MS in juvenile qingbo (*Spinibarbus sinensis*).

Exercise training has a positive effect on swimming performance in many fish (Pearson et al. 1990; Young and Cech 1993; Holk and Lykkeboe 1998; Liu et al. 2009; Li et al. 2010). This effect is due to an improvement in cardio-respiratory capacity (Farrell et al. 1991; Eme et al. 2009; Liu et al. 2009; Fu et al. 2011), an increase in muscle fibre size, an increased number of mitochondria (Davison and Goldspink 1977; Davie et al. 1986), and the enhanced activity of mitochondrial enzymes (Johnston and Moon 1980). Other studies, however, have shown that exercise training has no effect or even negative effects on respiratory capacity and swimming performance (Scarabello et al. 1992; Thorarensen et al. 1993; Gallaugher et al. 2001; Fu et al. 2011). The precise reasons behind these differing results are unknown but are generally hypothesised to be related to differences among fish species and in training protocols, training intensity and/or training duration. Exercise training protocols may be categorised as aerobic (endurance) or anaerobic (sprint) (Pearson et al. 1990; Liu et al. 2009). Endurance training protocols typically involve swimming at 1–3 body lengths per second (BL s^−1^) for extended periods of time (1–12 months), whereas sprint exercise training typically involves burst exercise, often achieved by chasing and performed daily for short time periods. Previous research has demonstrated that training with exhaustive chasing can produce a substantial effect on aerobic capacity in darkbarbel catfish (*Peltebagrus vachelli*) and American alligators (*Alligator mississippiensis*) (Eme et al. 2009; Liu et al. 2009). Accordingly, the second aim of this study is to test whether training with exhaustive chasing affects *U*_crit_, $\dot{M}{\text{O}}_{{ 2 {\text{active}}}}$, $\dot{M}{\text{O}}_{{ 2 {\text{active}}}}$ and MS in juvenile qingbo.

In a recent study, we found that swimming performance is limited primarily by the peripheral locomotor system at low temperatures and by the central cardio-respiratory system at high temperatures in some fish species (Pang et al. 2010; 2011). Because exercise training may improve either the central cardio-respiratory capacity or the peripheral locomotor metabolic capacity (or both capacities) depending on the fish species or experimental condition selected, we hypothesised that exercise training would have different effects on swimming performance at different temperatures in fishes. Thus, the third aim of this study is to test whether exhaustive chasing training has different effects on $\dot{M}{\text{O}}_{{ 2 {\text{active}}}}$, $\dot{M}{\text{O}}_{{ 2 {\text{active}}}}$ and *U*_crit_ at different temperatures (15 and 25 °C) in juvenile qingbo.

The qingbo is a species of cyprinid fish that usually inhabits areas with flowing water in southern China (Kong et al. 2007). It is a commercially important fish species and one of the most abundant fish species in the Three Gorges Reservoir (Duan et al. 2002). Changes in water temperature and velocity that may have occurred due to the construction of the Three Gorges Dam could have effects on the crucial physiological functions of all aquatic organisms (Bian and Chen 2006). Local adaptations of physiological functions within and among species are likely to play important roles in the responses to global climate change (Pörtner and Farrell 2008; Eliason et al. 2011). We therefore selected the qingbo as our experimental model and investigated the effects of temperature and exercise training on swimming performance in the context of the protection of local fish species.

## Materials and methods

### Experimental fish and holding conditions

Juvenile qingbo (3–5 g, *N* = 140) were obtained from local farmers in Chongqing, China. The fish were kept in a recirculating water tank (length × width × height, 1.5 m × 0.6 m × 0.5 m) system at Chongqing Normal University for 3 weeks before the experiments. During this period, the temperature of the fresh dechlorinated water was maintained at 20 ± 0.5 °C, the water oxygen content was kept above 7.0 mg L^−1^, the pH ranged from 6.5 to 7.3, and the ammonia-N ranged from 0.005 to 0.025 ppm. The photoperiod was maintained at 12-h light:12-h dark to simulate the natural light cycle. The fish were fed daily to satiation at 21:00 hours with a commercial diet.

### Experimental protocol

#### Temperature acclimation

After 3 weeks in the recirculating tank, 140 fish were randomly selected and divided into seven groups (including five temperature groups at 10, 15, 20, 25 and 30 °C; and two exercise training groups at 15 and 25 °C, respectively) of 20 fish and transferred to seven similar recirculating water tank systems. The water temperature was 20 °C when the fish were transferred, and it was then increased or decreased by 1 °C d^−1^ until it reached the prescribed temperature. The fish were maintained at the experimental temperature for 21 days. During the acclimation period, the fish were fed once daily to satiation.

#### Exercise training

Once the water temperature reached the prescribed values for the exercise training groups (15 and 25 °C), the fish were maintained at the experimental temperature for 7 days. Two groups of fish were then transferred from the recirculating holding tanks into a circular tank (outside diameter 104 cm and inside diameter 56 cm, with water speed approximately 65 cm s^−1^) for exercise training. The training of the two groups was performed daily at 15 and 25 °C, respectively. The training protocols followed Liu et al. (2009): in brief, fish were chased with a hand-net to exhaustive status in the circular container for approximately 20 min. The water temperature in the exercise training chamber was controlled to within ±0.5 °C. All fish were returned to the recirculating holding tanks after training. Training was conducted once per day at 1500 hours for 14 days.

The rearing conditions during the experimental period were consistent with those used during the temperature acclimation period. The fish were fed to satiation once daily at 2100 hours in the recirculating holding tanks during the training period. All of the fish fasted for 2 days before $\dot{M}{\text{O}}_{{ 2 {\text{rest}}}}$, *U*_crit_ and $\dot{M}{\text{O}}_{{ 2 {\text{active}}}}$ were measured.

### Measurements and calculations

After 21 days of temperature acclimation or 14 days of exercise training at 15 and 25 °C, 8 fish were selected from each group and subjected to $\dot{M}{\text{O}}_{{ 2 {\text{rest}}}}$, *U*_crit_ or swimming $\dot{M}{\text{O}}_{ 2}$ tests.

#### $\dot{M}{\text{O}}_{{ 2 {\text{rest}}}}$

Experimental fish that had previously fasted for at least 24 h were individually transferred to continuous-flow respirometer chambers [100 mL, for details see Fu et al. (2005)] and acclimated for 12 h. The oxygen consumption of individual fish was measured using an 11-chamber continuous flow respirometer. Up to 10 fish from any given experiment were studied, and one chamber without a fish acted as a control to represent background oxygen consumption. The following formula was used to calculate the $\dot{M}{\text{O}}_{ 2}$ (mg kg^−1^ h^−1^) of individual fish:

1 $$ {\dot{M}\text{O}}_{2} = \Updelta {\text{O}}_{2} \times \nu /m $$

where ∆O_2_ is the difference in oxygen concentration (mg L^−1^) between the experimental chamber and the control chamber (chamber without fish), *v* is the water flow rate in the experimental chamber (L h^−1^) and *m* is the body mass of the fish (kg). The dissolved oxygen concentration was measured at the outlet of the chamber with an oximeter (HQ20, Hach Company, Loveland, CO, USA). The flow rate of the water through the respirometer chamber was measured by collecting the water outflow from each chamber. The $\dot{M}{\text{O}}_{ 2}$ was measured 3 times at 2-h intervals, and the average was used as the $\dot{M}{\text{O}}_{{ 2 {\text{rest}}}}$.

#### U_crit_

A Brett-type swimming tunnel respirometer with a swim chamber with a 19.9-cm^2^ cross-sectional area was used to measure the fish’s *U*_crit_ [total volume 3.5 L; for details see Pang et al. (2010) and Li et al. (2010)]. Fish were individually transferred into the swim tunnel and allowed to recover for 1 h. The flow of aerated water through the respirometer was maintained continuously during this recovery period. The water temperature in the swimming chamber was controlled at 25 ± 0.2 °C using a water bath connected to a stainless steel heat exchanger. The water velocity was increased by 6-cm s^−1^ increments (approximately 1 BL s^−1^, where BL denotes body lengths) every 20 min until the fish fatigued. Fatigue was defined as the failure of the fish to move away from the rear honeycomb screen of the swimming chamber for 20 s (Lee et al. 2003a, b). The fish was then taken out of the swimming chamber, and measurements of body morphological parameters were taken to the nearest 0.1 cm after the measurement of *U*_crit_. *U*_crit_ was not corrected for the solid blocking effect because the cross-sectional area of the fish did not exceed 10 % of that of the swimming chamber. *U*_crit_ was calculated for individual fish using Brett’s equation (Brett 1964):

2 $$ U_{\text{crit}} = U_{\text{i}} + \, \left( {T_{\text{i}} /T_{\text{ii}} } \right)U_{\text{ii}} $$

where *U*_i_ is the highest speed at which the fish swam during the full-time period of the experiment (cm s^−1^), *U*_ii_ is the velocity increment (1 BL s^−1^; 8 cm s^−1^), *T*_ii_ is the prescribed period of swimming per speed (20 min) and *T*_i_ is the time that the fish swam at the final speed (min).

#### Swimming $\dot{M}{\text{O}}_{ 2}$

A small fraction of the water from the sealed respirometer was siphoned past the probe of an oximeter (HQ20, Hach Company, Loveland, CO, USA) in a cuvette thermoregulated with a water bath. The oxygen concentration (mg L^−1^) in the water was recorded once every 2 min. In open mode, the respirometer was supplied with fully aerated and thermoregulated water that circulated in a reservoir tank at a flow rate of 500 mL min^−1^. In closed mode, the $\dot{M}{\text{O}}_{ 2}$ (mg kg^−1^ h^−1^) of an individual fish during swimming was calculated from the depletion of oxygen according to the following equation:

3 $$ {\dot{\text{M}}\text{O}}_{ 2} = { 6}0\,{\text{slope}}\,{\text{Vol/}}m $$

where slope (mg min^−1^) is the decrease in the water oxygen content per minute. The slope was obtained from linear regressions of time (min) and water oxygen content (mg L^−1^) using a statistical program (Excel 2003). Only slopes with an *r* ^2^ > 0.95 were considered in the analysis. Vol is the total volume of the respirometer (3.5 L) minus the volume of the fish, and *m* is the body mass (kg) of the fish. The water oxygen content in the respirometer was never allowed to fall below 85 % oxygen saturation (Claireaux et al. 2006). The maximum $\dot{M}{\text{O}}_{ 2}$ was used as the value for $\dot{M}{\text{O}}_{{ 2 {\text{active}}}}$ during the *U*_crit_ test (only *T*_i_ > 10 min were considered in the analysis when the fish swam at the fastest speed).

### Data analysis and statistics

STATISTICA 6.0 (StatSoft, Inc., Tulsa, OK, USA) was used for the data analysis. All values are presented as the mean ± SE, and *P* < 0.05 was used as the level of statistical significance. The effects of the acclimation temperature on *U*_crit_, $\dot{M}{\text{O}}_{{ 2 {\text{rest}}}}$, $\dot{M}{\text{O}}_{{ 2 {\text{active}}}}$ and MS in the experimental tests were determined using a one-way analysis of variance (ANOVA). The ANOVA was followed by a least significant difference multiple comparison test if a statistical evaluation of a difference between the values shown by different acclimation groups was necessary. The effects of temperature and exercise training on *U*_crit_, $\dot{M}{\text{O}}_{{ 2 {\text{rest}}}}$, $\dot{M}{\text{O}}_{{ 2 {\text{active}}}}$ and MS were determined using a two-way ANOVA. The ANOVA was followed by a *t* test if a statistical evaluation of a difference between the values shown by the non-trained and trained fish at the same acclimation temperature was necessary. Nonlinear estimation was used if necessary.

## Results

### Effect of temperature on swimming performance

#### *U*_crit_

**Fig. 1:**
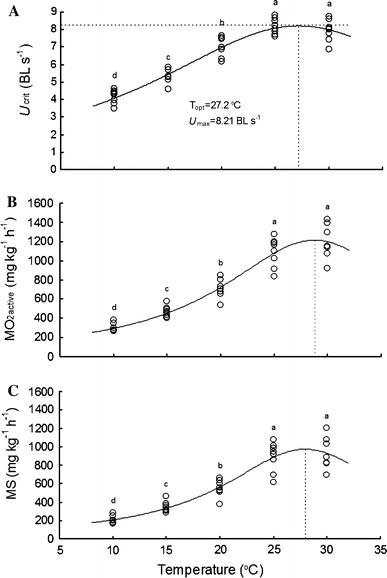
The relationships between temperature and *U*_crit_ (**a**), ${\dot{\text{M}}\text{O}}_{{ 2 {\text{active}}}}$ (**b**) and MS (**c**) in juvenile *Spinibarbus sinensis* were described by the equations *U*_crit_ = 8.21/{1 + [(*T* − 27.2)/17.0]^2^} (*R* ^2^ = 0.915, *P* < 0.001, *N* = 40); ${\dot{\text{M}}\text{O}}_{{ 2 {\text{active}}}} = 1 , 2 1 4. 2 9/\left\{ { 1 + \left[ {\left( {{\text{T}} - 2 8. 8} \right)/ 10. 6} \right]^{ 2} } \right\} \, \left( {R^{ 2} = 0. 9 1 1,\;P < 0.00 1,\;N = 40} \right)$; and MS = 972.67/{1 + [(T − 28.0)/9.34]^2^} (*R* ^2^ = 0.878, *P* < 0.001, *N* = 40); respectively. [*a, b, c, d* Values in each group without a common superscript are significantly different (*P* < 0.05)]

The *U*_crit_ of juvenile qingbo significantly increased with temperature over the range from 10 to 25 °C (*P* < 0.05), but no significant difference was found between 25 and 30 °C (Table 1). The relationship between *U*_crit_ and temperature (*T*) was described by the expression 8.21/{1 + [(T − 27.2)/17.0]^2^} (*R* ^2^ = 0.915, *P* < 0.001, *N* = 40) (Fig. 1). The optimal temperature for *U*_crit_ was 27.2 °C. The maximal *U*_crit_ at this temperature was 8.21 BL s^−1^ based on the regression equation (Fig. 1).

**Table 1:** Effects of temperature on several variables related to critical swimming performance in juvenile *Spinibarbus sinensis* (mean ± SE, *N* = 8)

Variables	Temperature (^o^C)				
	10	15	20	25	30
Body mass (g)	4.63 ± 0.19	4.37 ± 0.30	4.49 ± 0.34	4.36 ± 0.18	4.13 ± 0.21
Body length (cm)	6.01 ± 0.08	6.01 ± 0.09	6.06 ± 0.12	6.01 ± 0.06	6.01 ± 0.08
*U*_crit_ (BL s^−1^)	4.18 ± 0.14^d^	5.36 ± 0.14^c^	6.91 ± 0.20^b^	8.18 ± 0.16^a^	7.95 ± 0.21^a^
${\dot{\text{M}}\text{O}}_{{ 2 {\text{rest}}}} \left( {{\text{mg}} {\text{kg}}^{ - 1} {\text{h}}^{ - 1} } \right)$	93.0 ± 3.7^d^	121.4 ± 9.5^d^	152.3 ± 8.0^c^	200.3 ± 7.1^b^	267.8 ± 17.1^a^
${\dot{\text{M}}\text{O}}_{{ 2 {\text{active}}}} \left( {{\text{mg}} {\text{kg}}^{ - 1} {\text{h}}^{ - 1} } \right)$	296.4 ± 15.9^d^	468.7 ± 19.6^c^	701.6 ± 33.8^b^	1,088.5 ± 53.7^a^	1,195.3 ± 60.1^a^
MS (mg kg^−1^ h^−1^)	203.4 ± 15.0^d^	347.2 ± 20.2^c^	549.2 ± 31.5^b^	888.2 ± 56.3^a^	927.5 ± 57.8^a^

^a, b, c, d^ Values in each row without a common superscript are significantly different (*P* < 0.05)

#### $\dot{M}{\text{O}}_{{ 2 {\text{rest}}}}$

Body mass did not differ among the five temperature treatment groups before the experiment (Table 1). The $\dot{M}{\text{O}}_{{ 2 {\text{rest}}}}$ did not differ between the 10 and 15 °C treatments but increased significantly as the temperature increased from 15 to 30 °C (*P* < 0.05) (Table 1).

#### Swimming $\dot{M}{\text{O}}_{ 2}$, COT and COT_net_

**Fig. 2:**
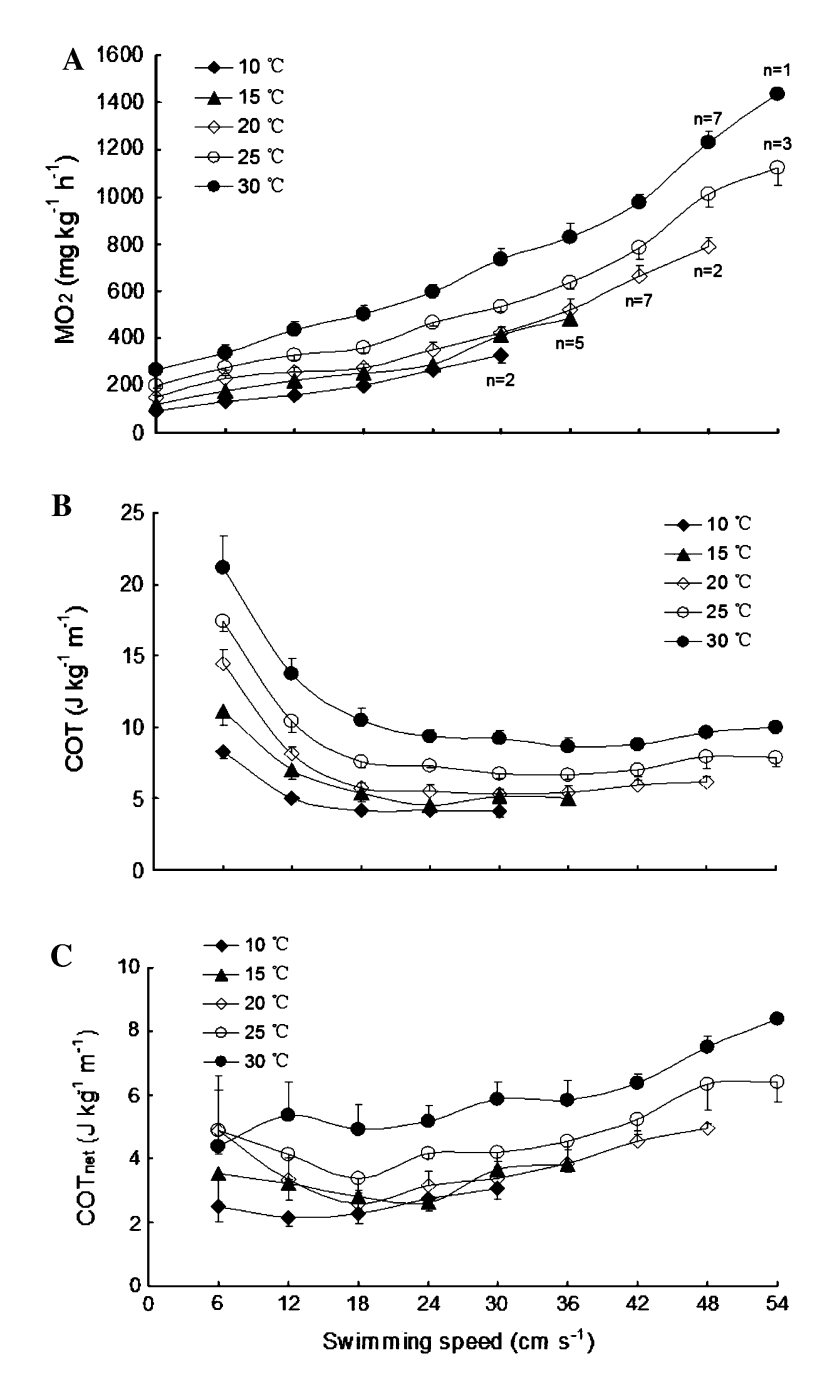
Effects of swimming speed on oxygen consumption rate $\left( {\dot{M}O_{2} } \right)$ (**a**), cost of transport (*COT*) (**b**) and net cost of transport (*COT*_*net*_) (**c**) of juvenile *Spinibarbus sinensis* at different temperatures [the $\dot{M}{\text{O}}_{ 2}$ values were converted to COT and COT_net_ (J kg^−1^ m^−1^) using an oxycalorific equivalent of 13.54 J (mg O_2_)^−1^ (Claireaux et al. 2006). Both swimming speed and temperature had significant effects on $\dot{M}{\text{O}}_{ 2}$, COT and COT_net_: two-way ANOVA, *P* < 0.05. A number of fish in each group did not attain the highest swimming speed] (mean ± SE)

Both temperature and swimming speed had significant effects on $\dot{M}{\text{O}}_{ 2}$, COT (cost of transport) and COT_net_ (net cost of transport) during the *U*_crit_ test (*P* < 0.05, Fig. 2a, b, c). The swimming $\dot{M}{\text{O}}_{ 2}$, COT and COT_net_ values of fish that were acclimated to high temperatures were significantly higher than those of fish acclimated to low temperatures at the same swimming speed (*P* < 0.05, Fig. 2a, b, c). The $\dot{M}{\text{O}}_{ 2}$ significantly increased with an increase in the swimming speed at each acclimation temperature (*P* < 0.05, Fig. 2a). The COT significantly decreased, then reached a plateau with an increase in the swimming speed at each acclimation temperature (*P* < 0.05, Fig. 2b). The COT_net_ significantly increased with an increase in the swimming speed at each acclimation temperature (*P* < 0.05, Fig. 2c).

#### $\dot{M}{\text{O}}_{{ 2 {\text{active}}}}$ and MS

The values of both $\dot{M}{\text{O}}_{{ 2 {\text{active}}}}$ and MS increased significantly as the temperature increased from 15 to 25 °C (*P* < 0.05), but there were no significant differences in these variables between the 25 and 30 °C treatments (Table 1). The relationships between ${\dot{\text{M}}\text{O}}_{{ 2 {\text{active}}}} = 1,214.29/\left\{ {1 + \left[ {\left( {T - 28.8} \right)/10.6} \right]^{2} } \right\} \, \left( {R^{2} = 0.911,\;P < 0.001,\;N = 40} \right)$ or MS and temperature were described as $\dot{M}{\text{O}}_{{ 2 {\text{active}}}}$ and MS = 972.67/{1 + [(T − 28.0)/9.34]^2^} (*R* ^2^ = 0.878, *P* < 0.001, *N* = 40), respectively (Fig. 1b, c). The optimal temperatures for $\dot{M}{\text{O}}_{{ 2 {\text{active}}}}$ and MS in juvenile qingbo were 28.8 and 28.0 °C, respectively.

### Effect of exercise training on swimming performance at different temperatures

#### *U*_crit_

**Fig. 3:**
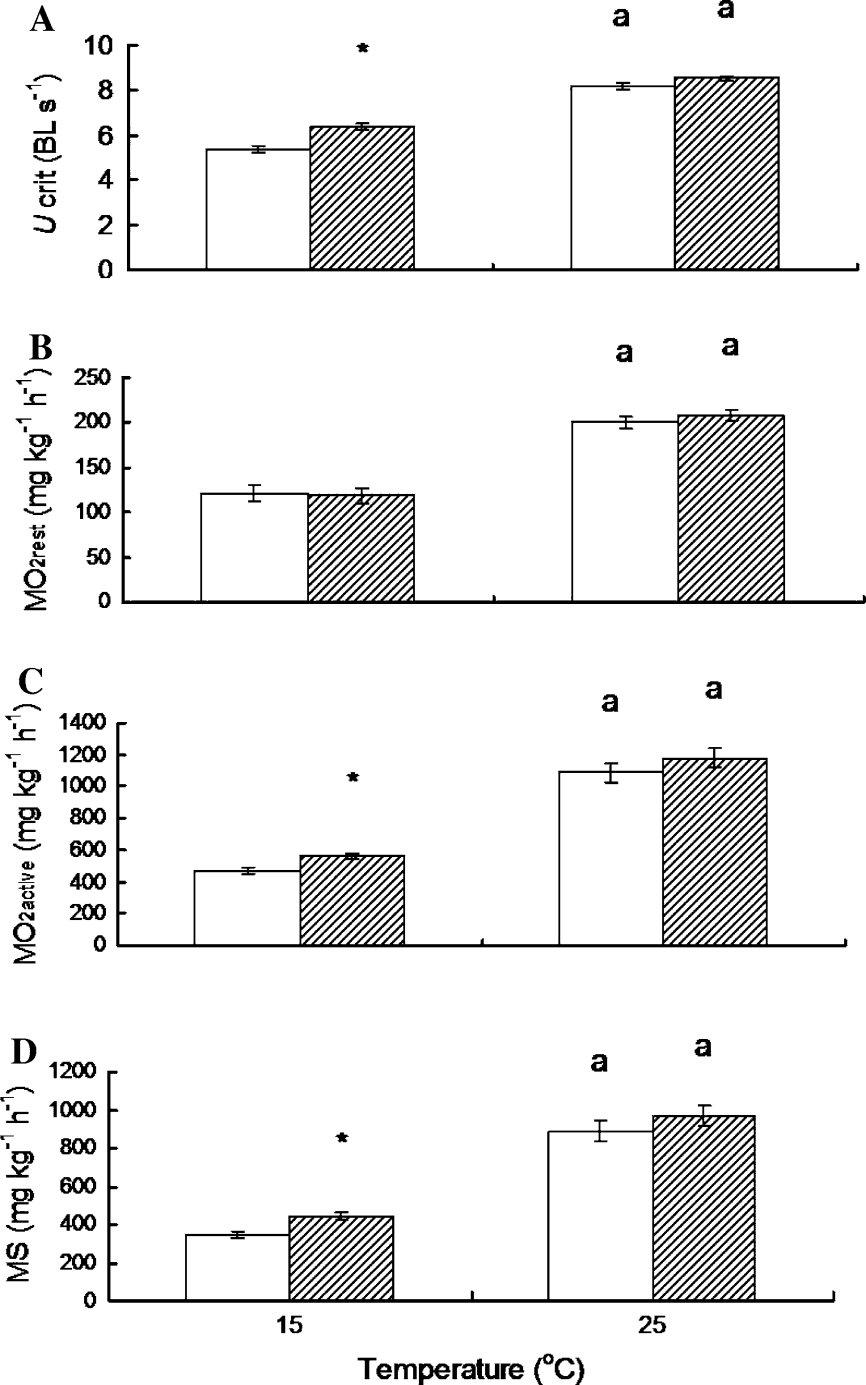
Effects of exercise training (non-trained *open bars*, trained *hatched bars*) and temperature (low and high temperature) on the *U*_*crit*_ (**a**), $\dot{M}O_{2rest}$ (**b**), $\dot{M}O_{2active}$ (**c**) and metabolic scope (*MS*) (**d**) of juvenile *Spinibarbus sinensis* (mean ± SE, *N* = 8). *Letters* indicate a statistically significant difference between the low (15 °C) and high (25 °C) temperature groups. *Asterisk*s denote a significant difference between non-trained and trained fish in a given temperature treatment group (*P* < 0.05) (the body mass and body length of the trained fish are 4.04 ± 0.09 g, 6.05 ± 0.06 cm and 4.07 ± 0.17 g, 5.99 ± 0.06 cm at 15 and 25 °C, respectively)

Exercise training had significantly different effects on *U*_crit_ values at the two acclimation temperatures (interaction effect, *P* < 0.05) (Fig. 3a). Exercise training resulted in a significantly higher *U*_crit_ at the low acclimation temperature (*P* < 0.05), but there was no difference in *U*_crit_ between the exercise-trained and control fish at the high acclimation temperature (*P* < 0.05) (Fig. 3a).

#### $\dot{M}{\text{O}}_{{ 2 {\text{rest}}}}$

Exercise training showed no effect on $\dot{M}{\text{O}}_{{ 2 {\text{rest}}}}$ at either acclimation temperature (Fig. 3b).

#### $\dot{M}{\text{O}}_{{ 2 {\text{active}}}}$ and MS

**Fig. 4:**
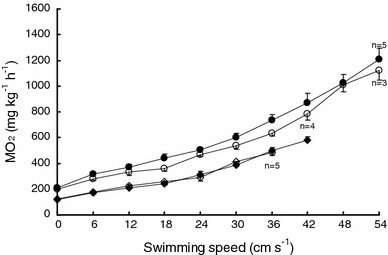
The effect of swimming speed on oxygen consumption rate $\left(\dot{M}\text{O}_{ 2} \right)$ at the low temperature (non-trained *open diamonds*, trained *filled diamonds*) and the high temperature (non-trained *open circles*, trained *filled circles*, there are no differences between non-trained and trained fish at the same swimming speed at either the low or the high temperature, *P* < 0.05) (mean ± SE)

Exercise training resulted in significant increases in both $\dot{M}{\text{O}}_{{ 2 {\text{active}}}}$ and MS at the low acclimation temperature (*P* < 0.05), but there was no significant difference in either variable between exercise-trained and non-trained fish acclimated to the high temperature (Fig. 3c, d). Exercise training had no effect on the swimming $\dot{M}{\text{O}}_{ 2}$ at either acclimation temperature (Fig. 4).

## Discussion

The present study investigated the effects of temperature and exercise training on swimming performance and the interaction effects produced by these factors in juvenile qingbo. We found that temperature had marked effects on *U*_crit_. The optimal temperature for *U*_crit_ was 27.2 °C, and the maximal *U*_crit_ at this temperature was 8.21 BL s^−1^. We also found that exercise training can improve the swimming performance of juvenile qingbo at a low temperature, but no such effect was found at a high temperature.

### Effect of temperature on swimming performance

Temperature is one of the most important physical factors affecting fish, with profound effects on many physiological and biochemical processes. Animals can adjust their physiological and biochemical processes to a new functional state if the environmental temperature changes (Kieffer 2000). It is a principal component of the final temperature preferendum paradigm that a relationship between the final temperature preferendum and the temperature at which centrally important processes take place at maximum efficiency (Lee et al. 2003b). In the present study, the relationship between *U*_crit_ and temperature was described as a bell-shaped curve with an optimal temperature for the *U*_crit_ of juvenile qingbo of 27.2 °C. This finding is similar to previously reported results for warm-water fishes, such as goldfish (*Carassius auratus*), largemouth bass (*Micropterus salmoides*), smallmouth bass (*Micropterus dolomieui*) and southern catfish. The optimal temperature for *U*_crit_ in these fishes varies between 25 and 30 °C (Hammer 1995; Zeng et al. 2009). In contrast, the optimal temperature for *U*_crit_ in cold-water fishes such as sockeye salmon (*Oncorhynchus nerka*) and cutthroat trout (*Oncorhynchus clarki clarki*) varies between 15 and 20 °C (Guderley 1990; Lee et al. 2003b; MacNutt et al. 2004). It has been suggested that the optimal temperature for *U*_crit_ in warm-water fish is higher than that for cold-water fish. According to the equations, the optimal temperatures for $\dot{M}{\text{O}}_{{ 2 {\text{active}}}}$ and MS in juvenile qingbo were 28.8 and 28.0 °C, respectively (Fig. 1b, c). The result showed that the optimal temperature (27.2 °C) for *U*_crit_ in juvenile qingbo was consistent with their optimal temperatures for $\dot{M}{\text{O}}_{{ 2 {\text{active}}}}$ and MS. The similar results have been previously documented in sockeye salmon (Lee et al. 2003b). It has been found that the optimal temperatures for *U*_crit_ and EPOC (excess post-exercise oxygen consumption) were 28.4 and 21 °C in southern catfish (Zeng et al. 2009, 2010). These results suggest that fish have different optimal temperatures for different physiological functions. Moreover, the optimal temperature for MS for the weaver sockeye salmon has increased with global climate warming (Eliason et al. 2011). Therefore, optimal temperatures are related to the biology of a species, its physiological functions and habits (Eliason et al. 2011).

In this study, the result showed that the *U*_crit_ of juvenile qingbo decreased as the water temperature decreased when the temperature was lower than the optimal temperature (Fig. 1a; Table 1). Our previous study found that the *U*_crit_ did not occupy all of the aerobic metabolic scope at low temperatures in qingbo (Pang et al. 2011). Hence, the decreased swimming capacity at low temperatures was due to the decreased metabolic capacity of the peripheral locomotor system rather than the respiratory capacity of the central cardio-respiratory system in qingbo. Previous studies also observed a decrease in enzyme activity in aerobic muscle fibres in the tropical freshwater fish *Oreochromis niloticus* and a decrease in adenosine triphosphate (ATP) in muscle tissues in killifish (*Fundulus heteroclitus*) at low temperatures. These decreases caused substantial reductions in swimming performance (Mwangangi and Mutungi 1994; Fangue et al. 2008). Furthermore, it has been found that EPOC was lower at low temperatures than that at high temperatures in some fish species (Lee et al. 2003a; Zeng et al. 2010). Thus, the decrease in anaerobic metabolic capacity may be a result of a decrease in *U*_crit_ at low temperatures. When the temperature was higher than the optimal temperature, the *U*_crit_ decreased with increases in water temperature due to the decreased aerobic metabolic scope (Lee et al. 2003b; Pörtner and Farrell 2008; Eliason et al. 2011). This study indicated a small decrease in *U*_crit_ at 30 °C. It is unfortunate that the maximum acclimation temperature in this study was not higher than 30 °C based on the range of temperature variation in qingbo habitat. It is worth considering including a higher acclimation temperature in a future study focused on global climate warming. The COT_net_ at a given swimming speed increased with increasing temperature (Fig. 1c), thus, the energy utilisation efficiency decreased with increasing temperature in qingbo. The decreased energy utilisation efficiency, breakdown of enzymes used in aerobic metabolism, decrease in dissolved oxygen and hampered arterial oxygen delivery at high temperatures produced more difficult respiratory conditions at high temperatures (Brett 1971; Farrell et al. 1996; Steinhausen et al. 2008).

### Effect of exercise training on swimming performance at different temperatures

In the present study, 14 days of exhaustive chasing training resulted in a 19 % increase in *U*_crit_ in juvenile qingbo at the low temperature (15 °C). This finding is similar to results previously documented for other fish species. In striped bass (*Morone saxatilis*), 60 days of exercise training from 1.2 to 2.4 BL s^−1^ resulted in a 30 % increase in *U*_crit_ (Young and Cech 1993, 1994). In darkbarbel catfish, exhaustive chasing training for 14 days caused a 16 % increase in *U*_crit_ (Liu et al. 2009). In goldfish, sustained training at 70 % *U*_crit_ for 48 h resulted in a 17 % increase in *U*_crit_ if the fish swam in hypoxic water (1.0 mg L^−1^) (Fu et al. 2011). In the present study, exhaustive chasing training produced a 20 % increase in $\dot{M}{\text{O}}_{{ 2 {\text{active}}}}$ and a 28 % increase in MS at 15 °C. Recently, we found that at 15 °C, postprandial swimming juvenile qingbo exhibited a 20 % increase in $\dot{M}{\text{O}}_{{ 2 {\text{active}}}}$ and a 31 % increase in MS compared to fasting swimming fish. This suggests that the aerobic swimming performance of juvenile qingbo was limited by the peripheral locomotor system rather than the respiratory capacity of the central cardio-respiratory system in qingbo at low temperatures (Pang et al. 2011). Thus, these results suggested that the increase in swimming performance after exercise training was at least partially due to the improved metabolic capacity of the peripheral locomotor system. The increase in the metabolic capacity of the peripheral locomotor system may be related to the increases in red muscle cell diameter and fibre number, muscle type proportions, number of mitochondria in muscle tissue and the activities of enzymes (Davison and Goldspink 1977; Johnston and Moon 1980; Davie et al. 1986; Sänger 1993; Farrell et al. 1991; Eme et al. 2009; Liu et al. 2009).

Although the exercise training having profound effects on all three variables at 15 °C, the most interesting finding of this study is that exercise chasing training has no effect on *U*_crit_, $\dot{M}{\text{O}}_{{ 2 {\text{active}}}}$ or MS in juvenile qingbo at a high temperature (25 °C). Other studies have also reported no effect of exercise training in other fish species. For example, in chinook salmon (*Oncorhynchus tshawyscha*), exercise training at 100 % *U*_crit_ had no effect on *U*_crit_ (Gallaugher et al. 2001). In goldfish, neither *U*_crit_ nor $\dot{M}{\text{O}}_{{ 2 {\text{active}}}}$ showed any change after sustained swimming training at 70 % *U*_crit_ for 48 h in normoxia (Fu et al. 2011). Whether training can positively affect *U*_crit_ may depend on the cardio-respiratory system, including cardiac growth, the increase in heart rate, certain cardiac enzymes, haematocrit, lamellar surface area, arterial oxygen content, skeletal muscle capillarity and tissue oxygen extraction (Gamperl and Farrell 2004). On the one hand, swimming performance may be limited by factors related to aerobic metabolic capability such as arterial oxygen delivery, a decrease in dissolved oxygen and imbalances in ion fluxes at high temperatures (Motais and Isaia 1972; Gonzalez and McDonald 1994; Farrell et al. 1996; 2008; Pang et al. 2010). On the other hand, Jain and Farrell (2003) reported that warm-acclimated rainbow trout accumulated more plasma lactate after a *U*_crit_ test and exhibited a poorer repeated swimming performance than cold-acclimated fish, and Lee et al. (2003a) found that salmon recover EPOC faster at a higher temperature. Thus, the stress of exhaustion could be shorter for fish living at a higher temperature, which provides less of a stimulus to remodel tissues. Therefore, the exercise chasing training cannot improve swimming performance in juvenile qingbo at high temperatures.

## Conclusion

Temperature had a significant effect on swimming performance in juvenile qingbo. The optimal swimming temperature for *U*_crit_ was 27.2 °C. Exercise training produced a significant increase in swimming performance at a low temperature (15 °C), but it had no effect on swimming performance at a higher temperature (25 °C). The different effects of exercise training on swimming performance at different temperatures may be related to changes in aerobic metabolic capability, arterial oxygen delivery, available dissolved oxygen, imbalances in ion fluxes and the intensity of the stimulus to remodel tissues with changes in temperature.
